# Solenoidal Micromagnetic Stimulation Enables Activation of Axons With Specific Orientation

**DOI:** 10.3389/fphys.2018.00724

**Published:** 2018-07-27

**Authors:** Laleh Golestanirad, John T. Gale, Nauman F. Manzoor, Hyun-Joo Park, Lyall Glait, Frederick Haer, James A. Kaltenbach, Giorgio Bonmassar

**Affiliations:** ^1^Athinoula A. Martinos Center, Massachusetts General Hospital, Charlestown, MA, United States; ^2^Harvard Medical School, Boston, MA, United States; ^3^Department of Neurosurgery, Emory University, Atlanta, GA, United States; ^4^Department of Neurosciences, Cleveland Clinic Lerner Research Institute, Cleveland, OH, United States; ^5^Ear, Nose and Throat Institute, University Hospitals Cleveland Medical Center, Case Western Reserve University, Cleveland, OH, United States; ^6^Frederick Haer Corporation, Bowdoin, ME, United States

**Keywords:** eddy currents, TMS, finite element method, microcoils, inductive stimulation, numerical modeling, neurostimulation

## Abstract

Electrical stimulation of the central and peripheral nervous systems - such as deep brain stimulation, spinal cord stimulation, and epidural cortical stimulation are common therapeutic options increasingly used to treat a large variety of neurological and psychiatric conditions. Despite their remarkable success, there are limitations which if overcome, could enhance outcomes and potentially reduce common side-effects. Micromagnetic stimulation (μMS) was introduced to address some of these limitations. One of the most remarkable properties is that μMS is theoretically capable of activating neurons with specific axonal orientations. Here, we used computational electromagnetic models of the μMS coils adjacent to neuronal tissue combined with axon cable models to investigate μMS orientation-specific properties. We found a 20-fold reduction in the stimulation threshold of the preferred axonal orientation compared to the orthogonal direction. We also studied the directional specificity of μMS coils by recording the responses evoked in the inferior colliculus of rodents when a pulsed magnetic stimulus was applied to the surface of the dorsal cochlear nucleus. The results confirmed that the neuronal responses were highly sensitive to changes in the μMS coil orientation. Accordingly, our results suggest that μMS has the potential of stimulating target nuclei in the brain without affecting the surrounding white matter tracts.

## Introduction

Implanted medical devices based on electrical stimulation such as cardioverter-defibrillators and pacemakers (Ellenbogen and Wood, 2008), spinal cord stimulation (Kreis and Fishman, 2009), and deep brain stimulation (DBS) (Montgomery, 2010) devices have become well-accepted therapeutic options to treat a wide variety of medical conditions. Electrical stimulation has considerable clinical impact in alleviating symptoms of an increasingly diverse range of neurological and psychiatric disorders including for example, cochlear (Gifford, 2013) and auditory brainstem implants for restoring hearing (Møller, 2006), DBS to treat symptoms of Parkinsonism (Benabid, 2003; Deuschl et al., 2006), cortical stimulation for epilepsy and depression (Howland, 2008; Morace et al., 2016; Williams et al., 2016), spinal cord stimulation for neuropathic pain (Lopez et al., 2016), and vagus nerve stimulation for epilepsy (Panayiotopoulos, 2011) and depression (O’Reardon et al., 2006; United States Congress Senate Committee on Finance, 2006), just to mention a few. More recently, electrical stimulation has also shown promise for the restoration of function of retinal implants to restore vision in the blind (Humayun et al., 2012; Shepherd et al., 2013; Zrenner, 2013; Ayton et al., 2014; Stingl et al., 2015).

**FIGURE 1:**
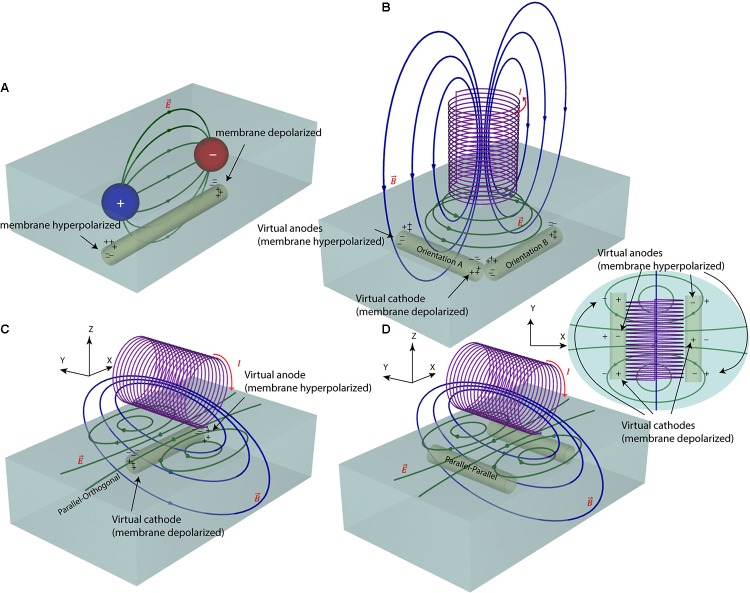
Electric and magnetic nerve stimulation mechanisms. **(A)** In electric nerve stimulation conductive electrodes are positioned in direct galvanic contact with the tissue. A DC (as in tDCS) or pulsed (as in DBS) voltage is applied between two electrode contacts to induce electric currents in the tissue. These currents follow a diffuse path from anode to cathode, hyperpolarizing neuron’s membrane under the anode and depolarizing it under the cathode. The current path, however, is diffuse and hard to control. **(B,C)** In magnetic nerve stimulation a time-varying electric current is passed through a coil, generating a time-varying magnetic field around the coil (as in TMS). According to Faraday’s law of induction, these time-varying magnetic fields induce a time-varying circular electric field in the tissue. The direction of this magnetically induced electric field depends on the orientation of the magnetic coil and thus, its stimulating effect on neurons can be better controlled. **(D)** For axons running with an orientation parallel to the axis of the coil, there will be no hyperpolarizing/depolarizing membrane net effect.

Although electrical techniques for neuronal stimulation have proven quite useful, they have several limitations that can be overcome by micro magnetic stimulation (μMS) which uses sub-millimeter coils. For example, for an electrode pair to generate currents it needs to be placed in contact with a conductive media (e.g., excitable tissue). Electric currents that are delivered by these electrodes diffuse and can spread to undesired areas adjacent to the structures being targeted, leading to unintended side-effects (Histed et al., 2009; Behrend et al., 2011; Licari et al., 2011; Weitz et al., 2015). A magnetic coil, on the other hand, can induce electric currents in the tissue from a distance (i.e., through an insulation layer). In nature these currents are closed-loop circular currents with a higher spatial focality (**Figure 1**). Furthermore, the fact that μMS coils can deliver stimulation while being insulated from the tissue increases their biocompatibility and compatibility with magnetic resonance imaging (considering no ferromagnetic material is present). Finally, as μMS coils can be positioned within or immediately adjacent to the neural tissue, the power needed to evoke neuronal activities is significantly reduced compared to techniques such as transcranial magnetic stimulation (TMS) which are designed to generate strong magnetic fields that pass through the skull and deliver stimulation to the cortical tissue.

**FIGURE 2:**
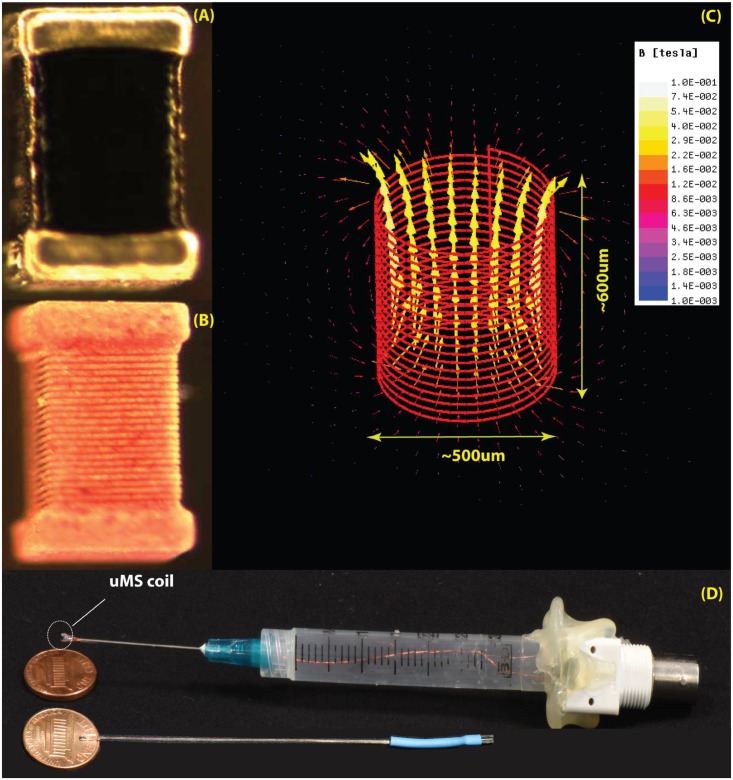
The μMS coils. **(A)** Image of the microcoil used in the experiments. **(B)** The outer layer of the conductor was chemically removed to expose the structure of the underlying solenoid. **(C)** Model of the coil with 21 turns of 6 μm gold microwire implemented in ANSYS Maxwell 18.0. The generated magnetic field is shown for a unit current of 1 A passing through the coil. **(D)** Top: the origin used in the experiments with the microcoil mounted on a syringe connected to a BNC connector. Bottom: a more compact model recently developed.

**FIGURE 3:**
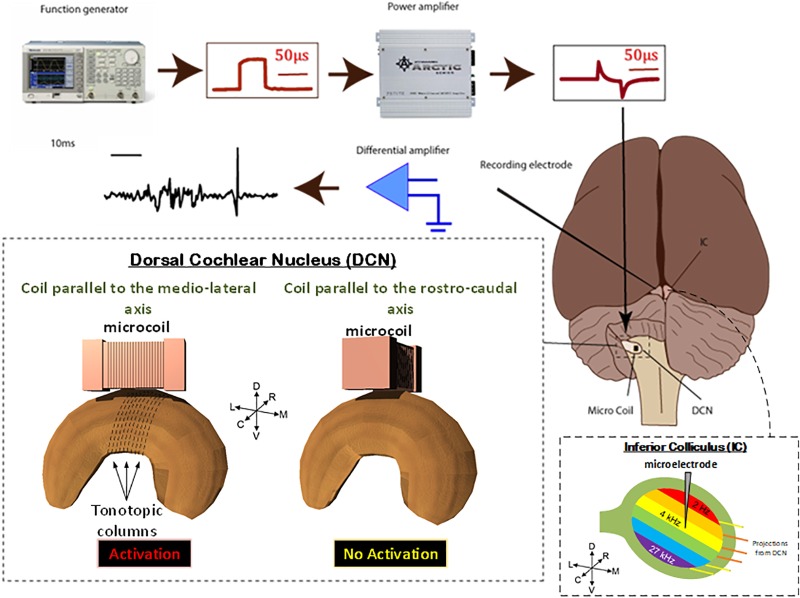
Animal preparation. Animals were anesthetized and the DCS and IC were surgically exposed. A recording electrode was placed into the IC and a μMS coil was positioned over the DCN. Stimulation was then applied to the coils, using a function generator and amplifier as electrophysiological data were simultaneously recorded from the IC. For each animal, the coil was first oriented along the medial–lateral axis of the DCN which evoked a strong response in the IC. The coil was then pulled up, rotated 90°, and positioned back on the same spot above the DCN. The latter rostrocaudal orientation of the coil evoked a much weaker response in the IC. To assure that the changes observed in the response were not due to disconnection of coil’s internal circuit during the rotation manipulation, we rotated the coil back to the medial–lateral orientation which again evoked a strong response from the IC. The stimuli were delivered to the DCN from microscopic stimulators with different orientations with respect to the long axis of the DCN: coil parallel to the medial–lateral axis will stimulate fibers in the rostrocaudal orientation (red).

**FIGURE 4:**
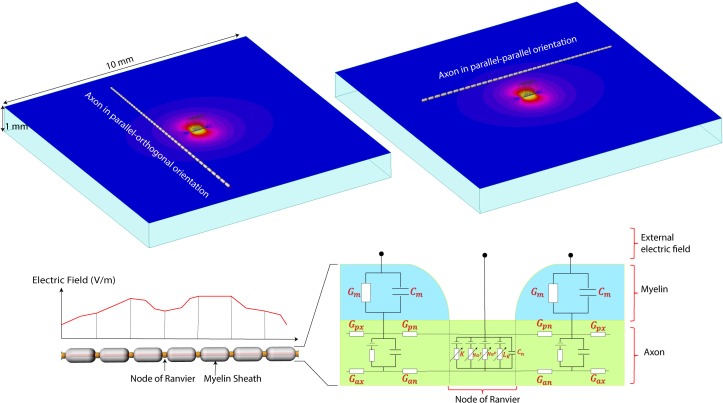
The NEURON model. **(Top)** A computational model of the micromagnetic coil suspended over a 10 mm × 10 mm × 1 mm slab of tissue. Electric and magnetic fields are calculated inside the whole volume of tissue. Field values on a transverse plane located 20 μm below the surface of the coil were exported to simulate the behavior of neurons. **(Bottom)** Model of the axon used in NEURON simulations. The parameters for the axonal conductance (Gax), the transmembrane conductance (Gm) and capacitance (Cm), the voltage-gated ion channel membrane conductance at the Node of Ranvier was adopted from Johnson and McIntyre (2008).

**FIGURE 5:**
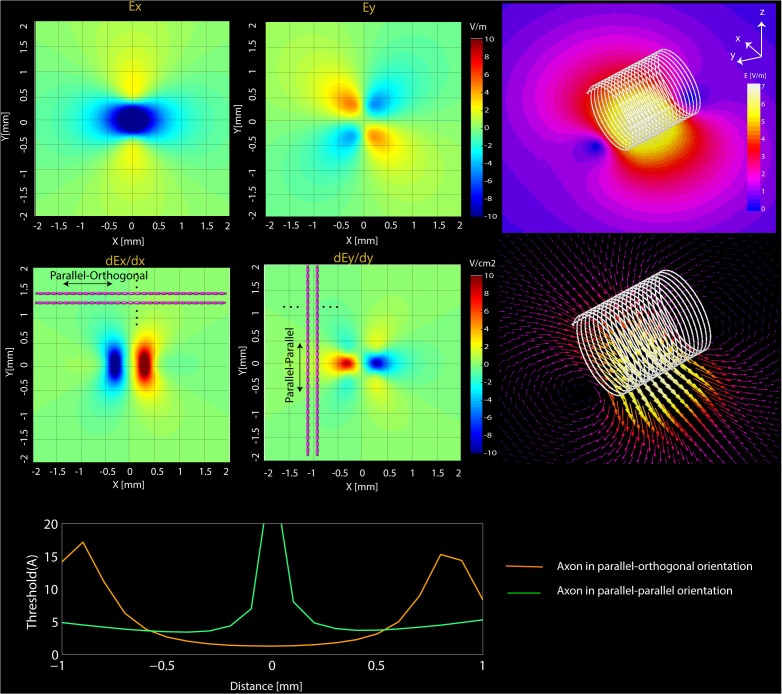
Simulations results. **(Top)** Computational results of the magnitude of Ex and Ey and their spatial derivatives (dEx/dx, dEy/dy) on transverse planes located at 20 μm below the surface of microcoil. **(Bottom)** NEURON estimated current threshold levels for the two orientations (orange-mediolateral and blue-rostrocaudal) at various distances from the axon.

**FIGURE 6:**
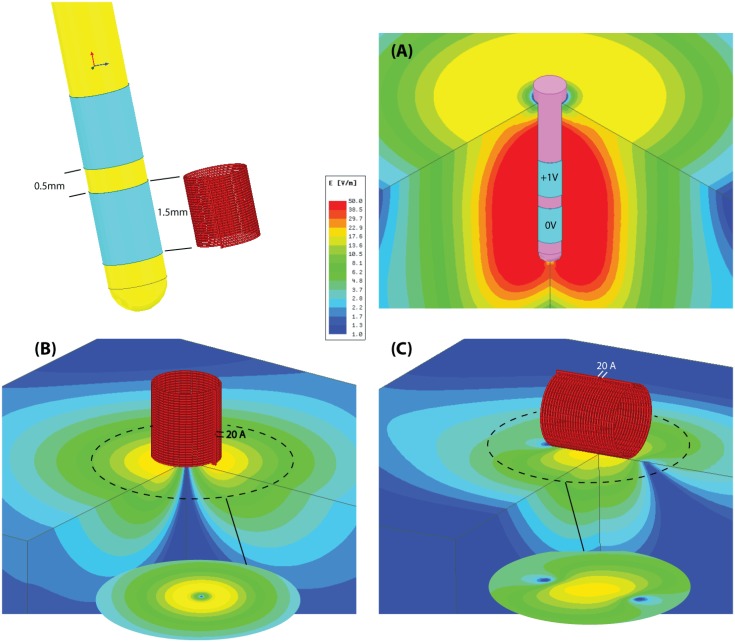
Simulation results. Schematic comparison of the distribution of electric field produced during electric and magnetic nerve stimulation. **(A)** A conventional DBS electrode pair in bipolar configuration (electrode length, diameter, and spacing mimics the Medtronic Lead 3389). The differential voltage between anode and cathode is set to be at the lowest limit of typical values (ranging from 1 to 5 V). **(B)** A μMS coil with dimensions matching the DBS electrode. The coil is fed with 20A similar to those used in our experiments and is perpendicular to the surface of the tissue. The electric field is symmetrical, but is confined to an annulus region under the coil. **(C)** A μMS parallel to the surface of the tissue. The electric field is mostly confined to a region beneath the coil and it’s asymmetric, leading to different neural activation thresholds depending on neurons’ axonal direction.

Our group recently demonstrated the feasibility of using μMS to elicit neuronal activation *in vitro* (Bonmassar et al., 2012), as well as the activation of neuronal circuitry on the system level *in vivo* (Park et al., 2013). As μMS is a novel technology, its mechanism(s) of nerve activation, induced field characteristics, and optimum topological features are yet to be explored. In this work, we performed numerical simulations to provide an insight into spatial distribution of μMS-induced electric fields, which in turn dictate the dynamics of nerve stimulation threshold changes with different axonal directionalities. Electromagnetic simulations were performed to estimate the magnetic flux 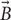 and the electric field 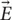 and its spatial gradient at different distances from the coil. These simulations were based on the actual coil prototypes built (**Figure 2**) and utilized in our animal experiments (**Figure 3**). The estimated 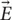 fields were then used in conjunction with the NEURON cable model to investigate the directional sensitivity of μMS (**Figures 4**, **5**). Finally, we performed *in vivo* experiments where we studied responses evoked in the inferior colliculus (IC) of rodents by applying μMS stimuli to the surface of animal’s dorsal cochlear nucleus (DCN). Specifically, we examined the IC responses to different coil orientations (**Figure 6**).

## Methods

### Electromagnetic Simulations

Numerical modeling has been long used to understand the phenomenology of field-tissue interaction in a wide variety of medical and diagnostic applications. Examples include use of electrostatic finite element modeling to predict the volume of activated tissue in electrical brain stimulation (McIntyre and Grill, 2001; Butson and McIntyre, 2006; Golestanirad et al., 2012b), eddy current modeling to assess the distribution of cortical currents in magnetic brain stimulation (Wagner T. et al., 2004; Wagner T.A. et al., 2004; Golestanirad et al., 2010, 2012c), and analysis of body exposure to low frequency magnetic fields and safety hazards due to motion of medical implants in magnetic fields (Condon and Hadley, 2000; Golestani-Rad et al., 2007; Golestanirad et al., 2012a). Recently, the role of numerical modeling has also been emphasized in safety assessment of MRI in patients with conductive implants (Clare McElcheran and Graham, 2014; Golestanirad et al., 2017a,b; McElcheran et al., 2017). The use of computational modeling to predict the response of neurons to external electric fields has been pioneered by eminent works of McIntyre and Grill (2001) and McIntyre et al. (2002, 2004) and followed by others (Wei and Grill, 2005; Woock et al., 2010; Golestanirad et al., 2012b, 2013). Electromagnetic simulations have also been successfully applied to quantify induced currents and assess the safety of transcranial magnetic brain stimulation (Wagner T.A. et al., 2004; Golestanirad et al., 2010, 2012c; Deng et al., 2013). In this work, we used ANSYS Maxwell (ANSYS, Canonsburg, PA, United States) which solves a modified T - Ω formulation of Maxwell’s Equations expressively designed for low-frequency calculations (Ren, 2002) using the finite element method (FEM). Simulations were performed with solenoidal μMS coils (500 μm diameter, 600 μm height, 21 turns, wire diameter 7 μm, carrying ∼20 amperes for a total current per turn = 420 AT). Coils were placed 20 μm above the surface of the tissue and were excited with a 70-kHz sinusoidal current. The tissue was modeled as a 10 mm × 10 mm × 1 mm slab of conductive material (σ = 0.13S/m). The ensemble of the coil-tissue system was enclosed in a 14 mm × 14 mm × 6 mm air box with Neumann boundary conditions applied to its outer faces which ensured that magnetic field was tangential to the boundary and flux did not cross it. ANSYS Maxwell was set up to follow an adaptive mesh scheme. A high-resolution initial tetrahedral mesh (60 μm) was seeded inside the tissue close to the coil. Maxwell generated a field solution using the specified mesh. It then analyzed the accuracy of the solution by calculating an energy value based on the error in the solution. The exact mechanism for evaluating the error varies by solution type. For eddy current solution, Maxwell uses ∇ × 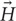 to find current density and then subtracts all input currents and other sources. For a perfect solution, the result would be zero, whereas for a real finite mesh the result would include some amount of residual current density. An energy value calculated from this residual current density is then used as the criteria to refine the mesh. An iterative process then will follow, which refines the mesh in each step until the energy error is below a user-specified value (1% in our case). The final solution had ∼630,000 mesh elements with edge length varying from 9 μm inside the tissue to 2 mm at the outer boundary the air box. The simulations converged after two adaptive passes which completed in 17 h on a Dell PowerEdge R730 with 16x32GB = 512GB of RAM, an NVIDIA K80 GPU and 28 cores (2xIntel Xeon CPU with each 14 cores) running 64-bit Windows Server 2012. Electric field values were then exported to MATLAB (The Mathworks, Inc., Natick, MA, United States) for smoothing and were used to simulate the response of neurons with different orientations below the coil.

### μMS Coil Orientations

In our previous work (Bonmassar et al., 2012) we showed that response of ganglion cells to μMS could be altered by changing the coil’s orientation. Specifically, we demonstrated that when the long axis of a solenoidal μMS coil was perpendicular to the surface of the excitable tissue (corresponding to **Figure 1B**), weaker neuronal responses were evoked compared to the case where the coil’s long axis was parallel to the surface of the tissue. Our surgical setup at the time, however, did not allow further examination of μMS directionality when the coil was parallel to the surface of the tissue. Theoretically, the μMS coil in a perpendicular orientation generates symmetric electric fields in the tissue underneath the coil, affecting axons with different orientations alike (see **Figure 1B**, axons with orthogonal orientations A and B experience similar electric field). This symmetry breaks down when the long axis of the coil is parallel to the surface of the tissue. In theory, the parallel μMS coil highly depolarizes axons that are located under its center and are orthogonal to its long axis (**Figure 1C**). We refer to this relative coil–axon orientation as the parallel–orthogonal orientation. In contrast, axons that are oriented parallel to the long axis of the coil (parallel–parallel orientation, **Figure 1D**) experience a reduced tangential electric field along with their length and should be minimally excited. We tested this hypothesis in NEURON simulations and in rodent experiments as described below.

### Neuron Modeling

A computational model of axons was built for simulation of neuronal activation for the three-dimensional electric fields obtained in the previous section. The parallel fiber axon model was assumed to have a diameter of 2 μm (Tolbert et al., 2004). Since the detailed information about the ion channels was not available, the ion channel properties were adopted from the double cable axon model of globus pallidus efferent axons (McIntyre et al., 2002; Johnson and McIntyre, 2008). In electrical or magnetic stimulation, the defining factor of axonal firing is the trans-membrane current at the nodes of Ranvier. When the transmembrane current is large enough to depolarize the membrane, an action potential initiates at the node and propagates both in the orthodromic and antidromic directions. Typically, the first node of activation is the node closest to the cathode in electrical stimulation or the coil in magnetic stimulation. In our neuronal simulation, the outgoing transmembrane current was calculated by the summation of the axonal current from the adjacent compartments in the compartmental model (Nagarajan et al., 1993; McIntyre et al., 2002; Carnevale and Hines, 2006). The axonal directional current density in each compartment is calculated by the multiplication of the axonal conductivity and the induced electric field in the axonal direction. Since the compartmental size of the double cable axon model is so small, the induced electric field in each compartment was assumed to be constant. The axons were assumed to be in a transverse plane 20 μm below the coil. The induced electric field at each compartment along the axon was obtained using bilinear interpolation of the electric field obtained in the previous section. A total of 41 axons were tested where the distance between each adjacent axon was set to 100 μm. Each axon was assumed to have 41 nodes of Ranvier, and the intermodal distance was set to 200 μm, and the center node was positioned at random distances from the coil. Regarding the orientation of the coil, we tested both the configurations where the axonal direction is parallel and orthogonal to the long axis of the coil. The software package NEURON was used to study the neuronal responses to the induced electric fields (Carnevale and Hines, 2006).

### Microcoil Construction

All microcoils were constructed to keep overall resistances below 5 Ω and inductances below 150 nH (4263B, Keysight Technologies, Santa Rosa, CA, United States) in order to ensure high stimulation efficacy. A multilayer inductor (ELJ-RFR10JFB, Panasonic Electronic Devices Corporation of America, Knoxville, TN, United States) was attached by soldering to two 34-AWG copper wires (Philmore Mfg., Rockford, IL, United States) with polyimide enamel inner coat and polyurethane overcoat. To insulate the tissue from the voltage applied and to protect against moisture, the microcoils were coated with an acrylic conformal coating (419C, MG = Chemicals, Burlington, ON, Canada) that offered high dielectric strength. The 419C Technical Data Sheet reports a thickness of 25 μm with an estimated variability of the dielectric thickness to be ± 5 μm. After curing (24 h), the insulation of the microcoil was tested by immersing the coils in a saline solution (0.9% of NaCl) and verifying that the resistance between the microcoil and an EGG electrode also dipped in the saline solution was greater than 5 MΩ (TX3, Tektronix, Inc., Beaverton, OR, United States). The microscopic stimulator was placed on the tip of a 23 AWG needle (Becton Dickinson, Franklin Lakes, NJ, United States) and the two wires inserted in the shaft/hub of the needle, and through the tip and barrel of a 3 ml syringe with the plunger that was removed and the flange end piece of the syringe was attached to a BNC connector by means of a glue gun, and electrically connected.

### Magnetic Stimulation

Two adult male Syrian golden hamsters were studied in this work. All procedures performed were approved by the Institutional Animal Care and Use Committee of the Cleveland Clinic, which adheres to the NIH Guide for the Care and Use of Laboratory Animals. In each experiment, μMS coils was mounted to a micromanipulator and manually positioned so that the coil was located just above the dorsal surface of the DCN, as described by Park et al. (2013). In our *in viv*o animal experiments, the μMS coils were driven by a generator (AFG3021B, Tektronix, Inc., Beaverton, OR, United States) connected to a 1,000-W audio amplifier (PB717X, Pyramid, Inc., Brooklyn, NY, United States) with a frequency band up to 70 kHz. The output of the amplifier was connected to a BNC splitter so that the signal sent to the μMS coil was monitored with an oscilloscope (DPO3012, Tektronix, Inc., Beaverton, OR, United States). Monophasic rectangular stimulation pulses with different pulse-widths and amplitudes were triggered by an analog A/D card (NI PCIe-6251, National Instruments), with an average rate of 2 Hz. The input pulse to the power amplifier and the corresponding output waveform of the power amplifier are shown in **Figure 3**. When referencing ‘stimulus amplitude’ in this paper, we indicate only the input pulse amplitude to the power amplifier. To prevent the carrying over effect from the previous trial, the order of the stimulation parameters (pulse amplitude and pulse-width) was randomized for each animal in addition to allowing 30 s resting periods between each 60 s.

### Electrophysiology

Recordings were conducted at multiple sites along the tonotopic axis of the central nucleus of the contralateral IC. This region was recognized by its sharp tuning properties and by the progression from high to low-frequency selectivities as the electrode was moved along the dorsoventral axis. Methods for recording and analyzing multiunit signals were similar to those described in previous studies (Manzoor et al., 2012, 2013). Signals were filtered and amplified using an Alpha Omega (SNR, Alpha Omega, Inc., Nazareth, Israel) preamplifier. Neural signals were digitized and read off the electrode channels using a National Instruments data acquisition board and customized software written in MATLAB Software was used to synchronize data collection with acoustic stimulus delivery for tuning curve and rate vs. tone level testing and with magnetic stimulus delivery. The software also allowed selection of stimulus parameters to test stimulus–response relationships.

### Acoustic Stimulation

Acoustic stimuli were needed for the dual purposes of characterizing the frequency tuning properties of recorded neurons to determine tonotopic coordinates of the IC recording electrodes, and also to examine effects of changing the acoustic stimulus conditions on IC responses. For both measures, we used 40 ms tone bursts (5 ms rise/fall times, 40 ms interstimulus intervals). For testing the tuning properties, we used a battery of 800 tone bursts varied in frequency from 3 to 32 kHz and in intensity from 6 to 96 dB SPL as previously described (Finlayson and Kaltenbach, 2009).

### Data Analysis

Samples of activity recorded from IC neurons were obtained from 100 to 150 repetitive stimuli and plotted as a function of time. The resulting time sweeps were used to compare responses during and following stimulation with the activity level recorded during the prestimulus period and to derive measures of several response characteristics, such as amplitude and magnetic coil orientation. Each of these measures was obtained for each of the parameters of stimulation that were tested and were compared with responses to baseline activities stimulation. Specifically, for each stimulation parameter (Amplitude and Orientation) baseline activities (15 ms before each stimulation pulse) were compared to stimulation response (15 ms following stimulation artifact; 17–32 ms). To facilitate comparisons, the absolute value of raw electrophysiological activities were summed for the baseline and stimulus–response periods for each stimulus delivered. Significant differences in responses for each parameter were compared using parametric ANOVA with Bonferroni corrections. In each analysis, responses were compared to their baseline activities and then tested relative to each amplitude or orientation.

## Results

We have explored the effects of coil orientation on the resulting stimulation capabilities both with numerical simulations and with animal studies.

### Numerical Simulations

The use of magnetic fields to induce electric fields or currents in the tissue from a distance is extremely inefficient from an energy standpoint. We hypothesize that much smaller energy than TMS may be required for neural stimulation at a microscopic level. One important difference between 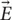 and 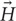 is that the magnitude of the latter is well-known to fall much more rapidly in space (e.g., quadratic vs. cubic law for an electric vs. magnetic dipole in empty space). Our hypothesis is based on the prediction by various activation models (Warman et al., 1992) that the gradient of the E field, is primarily responsible for neural stimulation. The FEM simulations confirm that the electric field gradient (i.e., 10^5^ V/m^2^) induced by a peak voltage of 35 V driven μMS in the physiological solution at the distances of 20 μm below the microcoil is comparable to the electric field gradient (i.e., 7.6 × 10^5^ V/m^2^) generated by a stimulation peak voltage of 5 V driven DBS electrode set (Astrom et al., 2015). In contrast, the electric field gradient sensitivity threshold for peripheral nerve stimulation in MRI (Koshtoiants Kh, 1957; Schaefer et al., 2000; Bencsik et al., 2007) is much smaller (Blake et al., 2014) (i.e., 150 V/m^2^) and can become near zero in quasi-uniform electric field modes such as in TMS (Barker et al., 1985; Bikson et al., 2013), given distance between the coil and the stimulated target region, which is referred to as the electric “farfield.” This farfield is hypothesized to produce stimulation through bends of the axon’s trajectory (Sheĭkh-Zade et al., 1987). Thus, the neuronal stimulation mode based on electric field gradient or “nearfield” is dominant for μMS, albeit farfield or combination of these two modes may also play a role for neurons further away.

The FEM simulations also predict that solenoidal μMS coils placed parallel to the surface of the tissue are capable of differentially activating neurons based on their axonal direction. It is established that neural activation function is proportional to the spatial derivative of the electric field along the axon’s axis (Roth and Basser, 1990). Our electromagnetic simulations predicted that the spatial derivative of electric field reached values up to three times higher for axons orientated in parallel–orthogonal orientation than for those oriented in parallel–parallel position (**Figure 5**, top). Similarly, NEURON simulations predicted that the axons whose direction is perpendicular to the long axis of the coil have a lower threshold compared to the axons parallel to the coil (**Figure 5**, bottom). The reason for lower threshold underneath the microcoil is that when the axons are parallel to the induced electric field the axonal activation is maximized since the activating function of an axon is the spatial derivate of the induced electric field along the axon. On the contrary, when the axonal direction is perpendicular to the induced electric field, the gradient of the electric field along the axonal direction becomes very small. Therefore, perpendicular coil orientation requires a much higher current threshold for the axonal activation underneath the coil. However, on the edges of the microcoil, there is a sudden change in the induced electric field due to its small size resulting in the increased activating function.

Importantly, and as demonstrated here, μMS provides a unique opportunity over electrical stimulation techniques in that neural interfaces can be constructed that take advantage of the orientation properties provided by magnetic stimulation. Namely, the construction of brain stimulation leads that maximally active the target tissue while mitigating the activation of fibers in the passage would have a significant advantage in DBS therapies, as the activation of fibers of passage represents the greatest side-effects for patients. Likewise, μMS coils could be used to provide more spatial resolution over existing electrical stimulation strategies, by designing interfaces that account for the orientation of the coils relative to the target tissue to be activated. This can be better appreciated from **Figure 6**, illustrating the schematic of the electric field distribution produced by (A) a conventional DBS electrode pair in bipolar configuration and (B,C) same-sized μMS coils in perpendicular and parallel positions. It can be observed from the figure that even for a DBS voltage as low as 1 V (typical values range from 1 to 5 V) the electric field produced by the electrode pair covers a large symmetric area containing both electrode contacts. The electric field of the μMS coil on the other hand, is more confined to the edges at the periphery of the coil when the coil is perpendicular to the tissue, and to the center of the solenoid when the coil is parallel to the tissue. Specifically, when the coil is in parallel position the induced electric field is asymmetric, indicating different sensitivity for neuron activation depending on their axonal direction. Moreover, we speculate that our modeling also brings up the notion that the mechanisms of action of magnetic stimulation may be fundamentally different from that of electrical stimulation. Specifically, the ionic movement of charge that ultimately results in neuronal activation operates differently between magnetic and electrical stimulation. In electrical stimulation, the current flow from pole to pole of the electrical stimulator while in magnetic stimulation the induced current flows as eddy currents relative to the magnetic fields. Electrical stimulation activates neural elements by operating on the electric potential of the extracellular matrix and manipulating the transmembrane potentials. In contrast, eddy currents act not only upon the extracellular matrix but also on the intracellular matrix as the magnetic stimulation fields penetrate the cellular compartments.

In general, μMS operates similarly to TMS generating time-varying magnetic fields and inducing electric fields in the brain, which can stimulate surrounding cortical or subcortical neurons (Walsh and Cowey, 2000), albeit at a microscopic scale. As TMS, which is presently the method of choice to investigate causal functional interactions across macroscopic brain regions, μMS can be used to investigate microscopic neuronal interactions at a cell level and as such can further the aim of developing innovative technologies to understand the human brain and treat its disorders.

### Animal Experiments

Recently it has been demonstrated that μMS is capable of eliciting neuronal activation in both retinal ganglion cells *in vitro* (Bonmassar et al., 2012) and IC neurons *in vivo* (Park et al., 2013). In the *in vitro* experiments, performed in a retinal cell preparation, it was demonstrated that neuron action potentials could be elicited by μMS. It was also demonstrated that neuronal activation was amplitude dependent, where higher amplitudes of simulation resulted in greater activation. Also, the orientation of the coils relative to the neural substrate resulted in a different activation pattern, where perpendicular orientations of the coil resulted in minimal activation and parallel orientations resulted in maximal activation. In the *in vivo* experiments, it was demonstrated that μMS of the DCN resulted in the generation of neuronal activities in the IC and in the cochlea. The *in vivo* experiments have recently been extended to the feline cochlea (Lee and Fried, 2017). Hence, μMS can elicit neuronal activation within an interconnected neural circuit and is not restricted to modulation of only local circuitry. Despite these results, some key issues need to be addressed before μMS can be further translated to chronic neuromodulation therapies, including the effect of coil orientation *in vivo* experiments.

**FIGURE 7:**
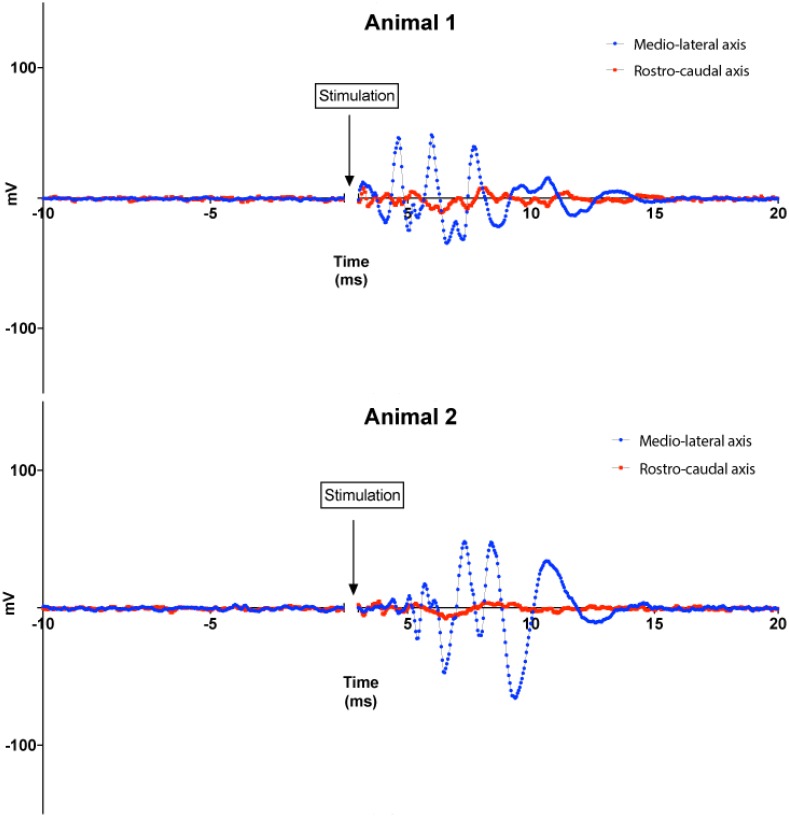
Animal experiments results. Top: responses of the IC to magnetic stimuli delivered to the DCN from microscopic stimulators with different orientations with respect to the long axis of the DCN. Blue lines: microcoil oriented parallel to the medial–lateral (tonotopic) axis of the DCN. Red lines: microcoil oriented perpendicular to the medial–lateral axis of the DCN. Recordings from two animals are shown for each orientation. Each curve represents the average of 100 trials performed for each coil orientation on each animal.

The microcoil prototypes we tested in this study reproducibly activated the brain, *in vivo*, in a tissue-appropriate manner consistent with the known microcircuitry of the DCN and projection patterns that link the DCN to the IC. Two adult male Syrian golden hamsters were studied, and all surgical procedures used to expose the DCN and IC were the same as previously described (Manzoor et al., 2012, 2013). The microcoils produced responses that were typically manifested in the contralateral IC as bursts or barrages of spike-like waveforms in the first 15–20 ms of the post-stimulus period (**Figure 7**). The responses to the microcoils placed just above but not in contact with the DCN surface produced well-defined activity that resembled the spike-like multiunit responses observed during sound stimulation (Kaltenbach and McCaslin, 1996; Kaltenbach and Afman, 2000).

In a set of experiments, we examined the effect of coil orientation on neuronal activation properties. Two different orientations of the microcoils were studied, one with the long axis of the μMS parallel to the long (medial–lateral or tonotopic) axis of the DCN (**Figure 3**, bottom), and the other with the long axis of the μMS coil parallel to the rostrocaudal axis of the DCN (i.e., parallel to the isofrequency bands) (**Figure 3**, bottom). Strong IC responses were observed for microcoil orientations parallel to the medial–lateral axis of the DCN, while weaker or absent responses for the orthogonal orientations (**Figure 7**, top). In animal 2 (**Figure 7**, bottom), both the medial–lateral orientations were different from the rostral–caudal orientation but were not different from animal 1.

An important aspect of our results that was unexpected was the dependence of the strength of the IC response on the rotational angle of the microcoil above the DCN. IC responses were vigorous when the long axis of the microcoil was parallel to the medial–lateral axis of the DCN but weak for micro-stimulator orientations parallel to the rostrocaudal axis. This difference implies contrasting levels of efficacy in stimulus–response coupling between the different micro-stimulator orientations. The simplest mechanism to explain this results is that micro-stimulator orientations parallel to the medial–lateral axis of the DCN more effectively excite the main output neurons of the DCN, the fusiform cells, which project to the contralateral IC (Beyerl, 1978; Ryugo and Willard, 1985; Cant and Benson, 2003). This greater effectiveness of activation may occur because any stimulation that fusiform cells receive may receive input directly from the fibers running along the tonotopic columns of the DCN that are activated by the microcoil in the medial–lateral axis orientation (Mugnaini et al., 1980; Blackstad et al., 1984; Manis, 1989; Kanold and Young, 2001) and potentiate the responses of fusiform cells to other inputs (Fujino and Oertel, 2003; Tzounopoulos et al., 2007). Activation of fibers running along the tonotopic column would be expected to be greater when the axis of the micro-stimulator is perpendicular to the axes of the tonotopic column, thus parallel to the medial–lateral axis, as shown by our numerical simulations results. At the present juncture, we have not yet elucidated precise targets of the stimulation of the DCN circuitry. Multiple neural populations in the ventral cochlear nucleus (VCN) also project to the IC and interact with the DCN circuitry as well. These polysynaptic pathways are potential targets and possibly underlie the generation of late-onset responses in the IC.

## Discussion

There are some limitations that currently limit the efficacy and safety of electrical stimulation. First, electric currents delivered by microelectrodes can spread to undesired areas adjacent to the targeted structures, leading to unintended side effects (Histed et al., 2009; Behrend et al., 2011; Licari et al., 2011; Weitz et al., 2015). For example, imprecise targeting of the subthalamic nucleus (STN) due to current spread to neighboring white matter tracts during DBS in Parkinson’s patients can lead to undesirable motor and sensory responses (Li et al., 2016). In this work, we show that unlike electrical stimulation μMS has the potential of being able to stimulate target nuclei in the brain without affecting the surrounding white matter tracts. Neuronal processes such as axons parallel to the direction of the electric current density 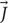 are depolarized or hyperpolarized depending on the direction and strength of 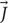, but the processes transverse to the 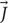 are not affected (Beurrier et al., 2001). Thus, the magnetic stimulation via μMS is capable of synaptically activating or inhibiting neurons in a spatially oriented manner. One aspect of the directionality of μMS was shown *in vitro* (Serano et al., 2015), where depending on the direction of the magnetic field flux the axon of the ganglion cell beneath the coil showed the generation of action potentials recorded by the patch clamping technique. In this work, we also expand this finding to our *in vivo* rodent model, showing for the first time, our ability to stimulate the brain stem of a rodent with a net sensitivity to the directionality of the magnetic flux. A similar μMS-orientation sensitivity was shown (Lee and Fried, 2017) in layer V pyramidal neurons (PNs) and the asymmetric fields arising from such microcoils did not simultaneously activate horizontally oriented axon Furthermore, μMS was shown to stimulate in confined narrow regions (<60 μm) cortical pyramidal neurons in brain slices *in vitro*, which helped to avoid the simultaneous activation of passing axons (Mehta and Oxenham, 2017). μMS coils were also surgically introduced 8–10 mm into the cochlea of anesthetized deafened felines (Lee and Fried, 2017), thus unresponsive to acoustic stimuli, and auditory responses were then recorded during magnetic stimulation. These experiments were aimed at showing that magnetic field steerability of μMS may solve the low-resolution stimulation shortcomings of the state-of-the-art cochlear implants that are limited by their ability to reproduce accurately pitch in music and speech in the presence of background noise, which instead may require as much as four times the number of channels currently available (Mehta and Oxenham, 2017). In the cochlea (Macherey and Carlyon, 2014) as well as in cortex (Matteucci et al., 2016) stimulation resolution is limited by the channel to channel cross-talk rather than electrode sub-millimeter size and spacing. μMS has shown the ability to selectively activate neurons by different orientations, thus a μMS coil in a single position can activate different neurons by rotation, thus increasing the spatial resolution.

Second, unlike electrical stimulation, μMS does not require direct galvanic contact with the tissue. For an electrode pair (**Figure 1A**) to generate current, it needs to be placed in direct contact with a conductive media (e.g., excitable tissue). In a bipolar electrode pair, the ‘anode’ or source (**Figure 1A**, plus sign) injects a current and hyperpolarizes the neuronal membrane toward more negative potential which can arrest the neural action, whereas the ‘cathode’ acts as a sink and depolarizes the axon membrane which could trigger an action potential (**Figure 1A**, minus sign). However, the metal electrode implanted in the tissue may lead to oxidation–reduction reaction at the electrode-tissue interface changing the pH of surrounding tissue which may provoke an immune response. Histopathology analysis has shown gliosis and spongiosis around the stimulation electrode track (Caparros-Lefebvre et al., 1994), which formed an encapsulation layer referred to as the “glial scar.” With μMS however, the solenoidal coil (**Figures 1B–D**) can induce a current from a distance, without placing a metal in direct contact with the tissue and new materials may allow for soft coils development (Wang et al., 2000). The pulsed current passing through the coil generates a time-varying magnetic field 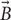 inside and in the space surrounding the coil. In the conductive tissue, this time-varying magnetic field 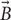, in turn, generates an orthogonal current density 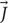 capable of evoking neuronal action potentials (**Figures 1B–D**), according to Faraday’s Law [i.e., $\frac{1}{\sigma }$∇×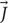 = −$\frac{\partial \vec{B}}{\partial t}$ in homogeneous isotropic medium where ∇× is the curl operator and σ is, the tissue conductivity, albeit the brain has tissues with anisotropic conductivities (Tuch et al., 1999)]. A number of studies have shown that the magnetically induced currents can directly excite axons as long as the spatial gradient of the induced electric field is strong enough to generate a transmembrane potential above the threshold (Roth and Basser, 1990; Basser and Roth, 1991; Pashut et al., 2011). The exact threshold depends on the axon’s geometry such as the diameter and its geometrical shape (Pashut et al., 2011), the pulse width (Basser and Roth, 1991), size and shape of the electrodes, etc. Furthermore, even though electric stimulation affects the myelinated neurons in the nodes of Ranvier, μMS can theoretically stimulate a myelinated axon anywhere within its length. Modulation of neuronal activation or inhibition can also be potentially achieved in μMS by driving specific waveforms (e.g., sharp rising edges followed by slowly falling dips, and vice versa), producing asymmetric induced current pulses in the tissue.

Finally, unlike electrical stimulation μMS does not require a charge-balanced stimulation waveform. In electrical stimulation, charge balancing is necessary to avoid excessive charge accumulation at the neural interface, and thus undesired stimulation and electroporation (Nduka et al., 2017). Electroporation occurs when the external electric field of the membrane potential of the cell exceeds a 0.2–1 V threshold, which leads to a change in the molecular structure of the membrane, and a subsequent membrane perforation with pore formation increasing the membrane permeability to ions, and molecules (Chen et al., 2006). Electroporation with a transmembrane potential of approximately 1 V could cause necrosis, due to membrane rupture and the subsequent cytoplasmic contents leakage (Sale and Hamilton, 1968; Neumann and Rosenheck, 1972; Crowley, 1973). In μMS, no net charge is transferred from the electrode into tissue since neither sinks nor sources are present when a current density 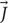 is induced by the time-varying magnetic field. The current density in the tissue 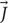 is a rotating field that mirrors the current direction in the coil (**Figure 1B**). Because the induced electric field is a solenoidal or incompressible vector field in three dimensions, μMS does not suffer from charge buildup (Bonmassar et al., 2012).

Despite these specific limitations, electrically based DBS has been tremendously successful. However, the application of μMS could mitigate some of the challenges of these limitations. Theoretically, and supported by limited data (Bonmassar et al., 2012), it is possible that specific orientations of magnetic fields relative to different neural substrates may result in differential neuronal response patterns. If demonstrated to be a valid property of magnetic stimulation, this would open the possibility of custom designing μMS coils in a way to maximize the stimulation of the intended target and minimizing the activation of unintended targets. In the case of DBS for movement disorders, the primary cause of side-effects is the unintended activation of fibers of passage. Namely, with STN stimulation the activation of the internal capsule, adjacent and lateral to the STN, or activation of the medial lemniscus fibers, medial to the STN, can cause muscle contracts or paresthesia respectively. Even if therapeutic efficacy is seen in a patient, it is possible that activation of these fibers of passage can limit the ultimate therapeutic effect, as the threshold of the side-effect may be less than the threshold for therapeutic benefit. The unintended activation of fibers of the passage is not unique to STN stimulation, as the unintended activation of the internal capsule is also seen with DBS of the ventral intermediate nucleus of the thalamus and globus pallidus internus, which are the other primary targets for DBS therapy for Parkinson’s disease. Hence, if it is demonstrated that orientation of magnetic fields has differential effects on the activation of axonal fibers, one can propose to custom design μMS coils to take advantage of this unique property. As this property is not directly achievable with electrical stimulation based technologies, it would provide a new avenue to improve outcomes and mitigate side-effects beyond the other limitation previously discussed between electrical and magnetic stimulation based approaches.

## Conclusion

Microscopic magnetic stimulation (μMS) could potentially become the pacemaker and brain stimulator of the future with their contactless ability to deliver the neuronal stimulation needed for therapeutic efficacy in patients with Parkinson’s disease, epilepsy, in need of implantable cardioverter-defibrillators or pacemakers, and so forth. Due to recent advancements in micro-machining technologies, we can now utilize manufactured inductors (or coils) constructed on the sub-millimeter scale to produce magnetic fields. Such coils would offer several advantages over classical electrical and TMS techniques. Unlike TMS coils, the coils are sub-millimeter in size and can be placed within or near a neuronal substrate, increasing spatial resolution and reducing the power needed to evoke neuronal activity. Moreover, because the coils are not in direct contact with the tissue and no current is directly injected into the tissue, they may overcome the inflammatory tissue encapsulation and mitigate charge buildup issues inherent in traditional electrical stimulation technologies. Our data indicate that these microcoils can activate neuronal activity with high degrees of spatial and temporal resolution and that the orientation of the coils relative to the tissue activated can be used to activate specific elements optimally and to avoid the activation of others. Future work will concentrate on developing specific neural models of the target structures to quantify the parameters of μMS for directional stimulation, and include more animal models to establish the statistical features of the neural response.

## Ethics Statement

This study was carried out in accordance with the recommendations of Public Health Service (PHS) Policy on Humane Care and Use of Laboratory Animals and the Animal Welfare Act. The protocol was approved by the institutional animal care and use committee (IACUC) Committee of the Cleveland Clinic.

## Author Contributions

LaG, JG, JK, and GB conceptualized the study; provided support and guidance with data interpretation. H-JP designed and performed the NEURON analyses with support from LaG and JK. NM and LyG designed and performed the animal experiments with support from JK and H-JP. LaG and GB contributed electromagnetic data analysis and visualization software. FH built the μMS coils. LaG wrote the manuscript, with contributions from GB and comments from all other authors.

## Conflict of Interest Statement

JG provided consulting services for Alpha Omega Co. USA, Inc. and author FH was employed by the company FHC, Inc. The remaining authors declare that the research was conducted in the absence of any commercial or financial relationships that could be construed as a potential conflict of interest.
